# Quackery Rampant

**Published:** 1858-01

**Authors:** 


*Quackery Rampant.*—In spite of the hardness of times, quackery flourishes, with unabated vigor. People who are too poor to pay their just debts can afford to throw away dollars at a time for a bottle of stuff which perhaps costs the fabricator as many pennies, and though they fail of receiving the benefit which it promised, will seize with avidity on the next that offers, regardless of cost. I believe this branch of the business community is the only one which has not felt the pressure and been obliged to suspend. Lately, some of the lowest kind have been throwing into every house a circular of the most filthy character, a number of which I have encountered in my rounds. It is quite a common thing to be insulted by a boy putting in your hand a book of cases, certificates of cures, &c., with an almanac attached. This is a thing that should be suppressed by the police. Besides this, column after column of almost every paper you pick up is filled with their advertisements, and we might verily believe it impossible to die with so much positive proof of astonishing cures, &c. Apart from all other considerations, how many are actually injured by these nostrums! Again, how cruel is it to arouse within the breast the wildest hopes of cure in the case of some cherished relative or friend, only to be dashed down again, and deeper, owing to the height to which they have been raised.—*Ib.*

